# Ga_0.35_In_0.65_ N_0.02_As_0.08_/GaAs bidirectional light-emitting and light-absorbing heterojunction operating at 1.3 μm

**DOI:** 10.1186/1556-276X-9-37

**Published:** 2014-01-17

**Authors:** Faten Adel Ismael Chaqmaqchee, Naci Balkan

**Affiliations:** 1 University of Koya, Faculty of Science and Health, Kurdistan Region, Koya KOY45, IRAQ; 2 School of Computer Science and Electronic Engineering, University of Essex, Colchester CO43SQ, UK

**Keywords:** THH-VCSOA, Bidirectional, GaInNAs, Amplification

## Abstract

The Top-Hat hot electron light emission and lasing in semiconductor heterostructure (HELLISH)-vertical-cavity semiconductor optical amplifier (THH-VCSOA) is a bidirectional light-emitting and light-absorbing heterojunction device.

The device contains 11 Ga_0.35_In_0.65_ N_0.02_As_0.08_/GaAs MQWs in its intrinsic active region which is enclosed between six pairs of AlAs/GaAs top distributed Bragg reflectors (DBRs) and 20.5 pairs of AlAs/GaAs bottom DBR mirrors. The THH-VCSOA is fabricated using a four-contact configuration. The wavelength conversion with amplification is achieved by the appropriate biasing of the absorption and emission regions within the device. Absorption and emission regions may be reversed by changing the polarity of the applied voltage. Emission wavelength is about 1,300 nm and a maximum gain at this wavelength is around 5 dB at *T* = 300 K.

## Background

Vertical-cavity semiconductor optical amplifiers (VCSOAs) at 1.3 μm are key photonic components in optical communication systems [1-4]. Dilute nitride III-V alloy semiconductors and in particular GaInNAs/GaAs quantum well (QW)-based VCSOAs were originally proposed as replacements for GaInAsP/InP QWs due to its reduced temperature sensitivity and inherent polarization insensitivity [5,6]. In addition, their growth on GaAs and their integrability with GaAs/Al(Ga)As distributed Bragg reflectors (DBRs) allowed them to be considered as the active region in 1.3-μm vertical-cavity devices. In this article, a novel VCSOA based on the hot electron light emission and lasing in semiconductor heterostructure (HELLISH) as an alternative to conventional VCSOAs is investigated [7]. Spontaneous emission of ultra bright HELLISH has been previously reported and demonstrated by us [8,9]. The simple bar HELLISH-VCSOA [10] and Top-Hat HELLISH-VCSOA [11] structures with GaInNAs/GaAs quantum wells in the active region are designed to operate in the 1.3-μm wavelength region.

In this work, we demonstrate for the first time, optical amplification at wavelength *λ* ≈ 1.3 μm in electrically pumped THH-VCSOA devices. We measured the photoluminescence (PL) and electroluminescence (EL). By combining the two measurements, we obtained the electrophotoluminescence (EPL) signal from which the light amplification is obtained. At a temperature of *T* = 300 K, maximum gains were achieved when voltages of 40, 60, and 80 V were applied.

## Methods

**Figure 1:**
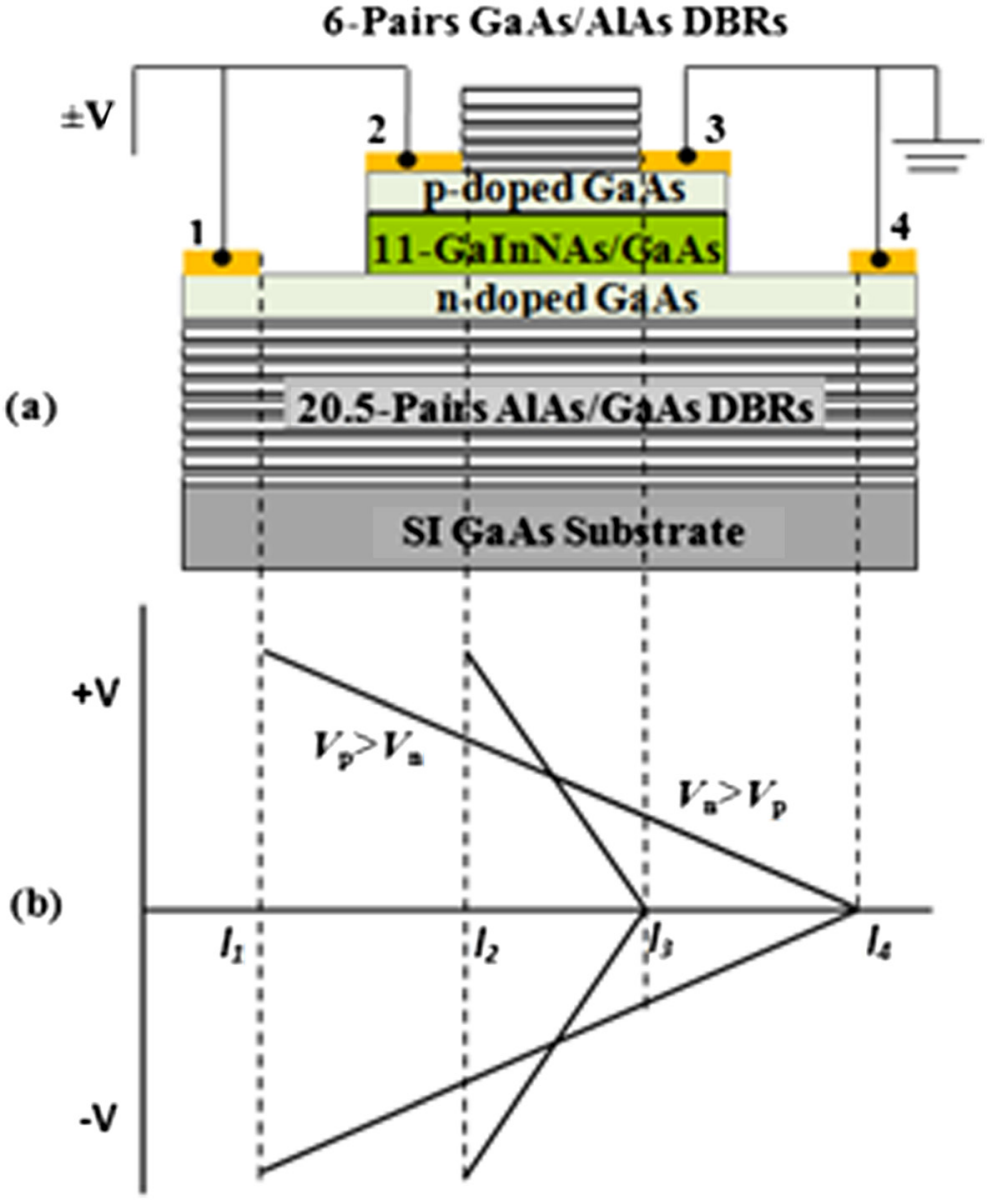
**Schematic diagram of (a) THH-VCSOA structure and its contact configuration and (b) potential distributions along p-channel and n-channel.** In the region of *V*_p_ > *V*_n_, the device is forward biased, while in the region of *V*_n_ > *V*_p_, the device is reverse biased.

The device of THH-VCSOA with the code VN1520 was grown by molecular beam epitaxy (MBE) on a semi-insulating GaAs substrate. Figure 1a shows the sample structure. Eleven Ga_0.35_In_0.65_ N_0.02_As_0.08_/GaAs QWs were used in the active region to supply enough gain at a wavelength of around 1.28 μm. The active region is within a micro-cavity which was formed by growing DBRs below and above the active region. Top and bottom DBRs have 6 and 20.5 pairs of AlAs/GaAs, with mirrors yielding calculated reflectivities of 0.6 and 0.99, respectively. The device was fabricated by selective etching to have a p-channel of length 0.6 mm and an n-channel of length 1 mm. Under normal operational conditions, contacts 1 and 2 are biased with either positive polarity (+V) or negative polarity (-V) while contacts 3 and 4 are both connected to the ground.

When the device is biased with (+V), as shown in Figure 1b, the potential near contact 2 (*I*_2_) is higher in the p-channel than in the n-channel (*V*_p_ > *V*_n_). This forward-biased region operates as a light emitter. In contrast, near contact 3 (*I*_3_), *V*_p_ < *V*_n_ and this region is effectively reverse biased, which forms the absorption section. Thus, the device can absorb light with photon energies of *hv*_*0*_, where *hv*_*0*_ > *E*_*g*_ and emit light with photon energies of *hv*_*1*_ *~ E*_*g*_. The polarity of the applied bias can be interchanged leading to the reversing of the absorption and emission regions.

The emitted light from the sample surface was collected and dispersed using a cooled photo multiplier and monochromator assembly. The output signal was filtered using an EG&G 162 boxcar averager with gated integrator. An Argon laser of wavelength *λ* = 488 nm, using variable powers, is used as the light source in the absorption experiments. External bias was applied in a pulsed mode between contacts 1 and 4, and 2 and 3 of the top-hat-shaped device. The device resistance depends on the device dimensions and can be as high as 1.0 KΩ in devices with long channel lengths. The applied voltage pulses were 50-μs wide with a repetition time of 10 ms defining a duty cycle of 5 × 10^3^.

## Results and discussion

**Figure 2:**
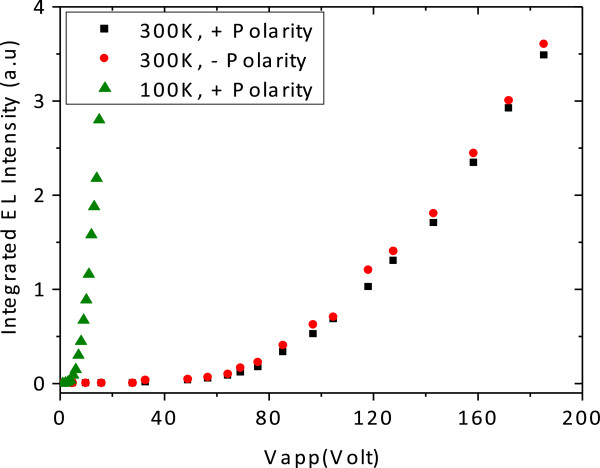
**Integrated electroluminescence intensity of bidirectional field effect light-emitting and light-absorbing heterojunction device (for both voltage polarities).** Temperatures of *T* = 100 and 300 K.

Figure 2 shows integrated EL intensity as a function of applied voltage for both voltage polarities. The EL was measured using voltage pulses of widths and repetition times of about 50 μs and 9 ms, respectively, to avoid excessive Joule heating. It is clear that the light intensity is independent of the polarity. The threshold voltages *V*_th_ of the bidirectional device are *V*_th_ approximately 50 V at *T* = 300 K and *V*_th_ approximately 4 V at *T* = 100 K.

**Figure 3:**
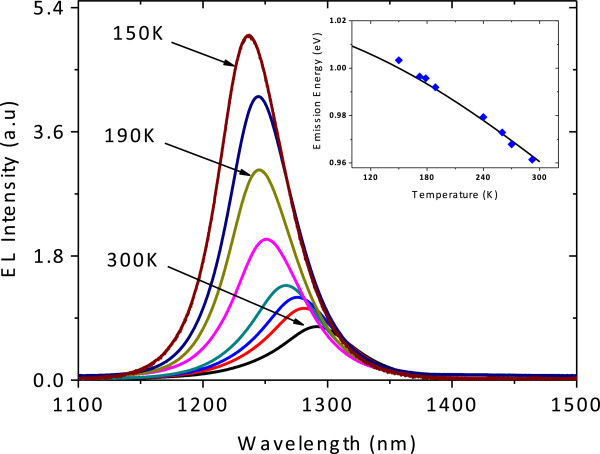
**EL spectra of bidirectional THH-VCSOA-based GaInNAs/GaAs structures at different temperatures.** The inset shows the temperature dependence of the peak energy (filled squares) compared with the Varshni equation (dotted lines).

Figure 3 shows the EL emission spectra as a function of temperature. The peak wavelengths at *T* = 150 and 300 K are around *λ* = 1,236 and 1,288 nm, respectively. Theoretically, a red shift of the active material peak wavelength with temperature at a rate of 0.38 nm/K is predicted. We compare the experimental peak emission energy versus the temperature plot with the Varshni equation:

$$\;E_{g}\left(T\right)=E_{0}-\alpha T^{2}/\left(T+\beta \right),$$

where *E*_0_ and *E*_*g*_(*T*) are the bandgaps at *T* = 0 K and at a finite temperature of *T*, respectively and *α* and *β* are around 4.8 × 10^-4^ eV · K^-2^ and 284 ± 167 K, respectively [12,13].

**Figure 4:**
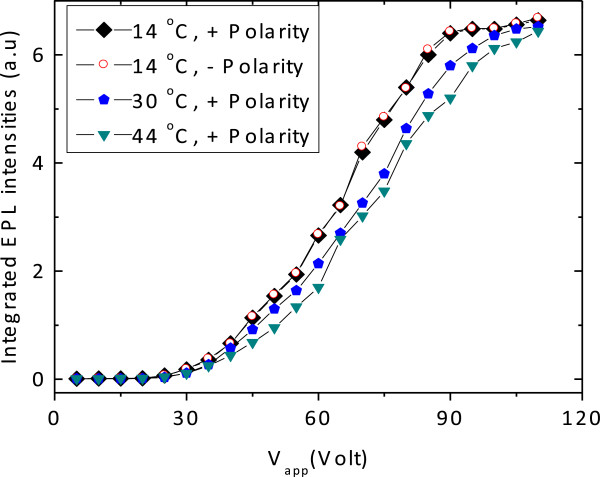
Temperature-dependent amplified signal of bidirectional THH-VCSOA structure.

The device was mounted on a temperature-controlled holder at varied temperatures. External voltage pulses up to 110 V were applied between the diffused contacts and the integrated EPL intensities of the THH-VCSOA are measured as a function of bias voltage with the photo-excitation power was kept constant at around 17 mW. In Figure 4, we show the peak intensities of EPL signals for both positive and negative polarities at *T* = 14°C and for positive polarity at temperatures of *T* = 30 and 44°C.

**Figure 5:**
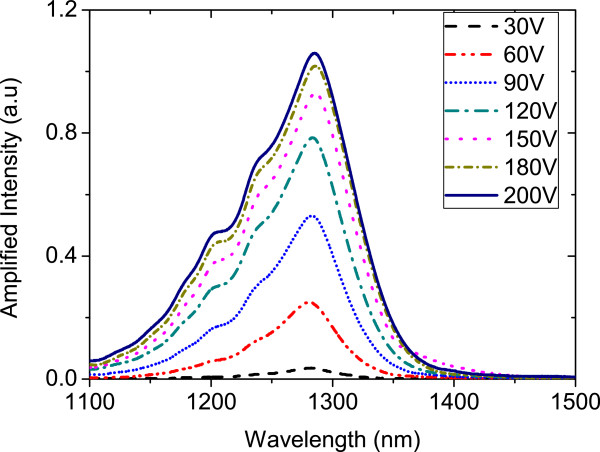
**Amplified intensity as a function of applied voltages between 30 and 200 V at *T*** **= 300 K.**

Amplified spectra are plotted as a function of applied voltages in Figure 5. It is clear from the figure that as the applied voltage increases, the integrated intensity increases with the emission peak at around 1,280 nm.

**Figure 6:**
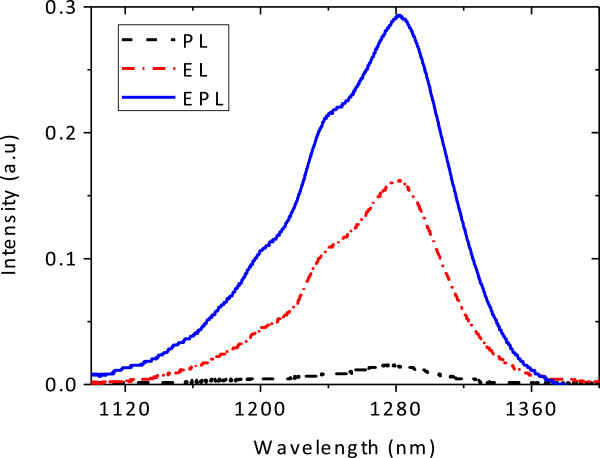
**PL, EL, and EPL spectra of THH-VCSOA at *T*** **= 300 K.**

**Figure 7:**
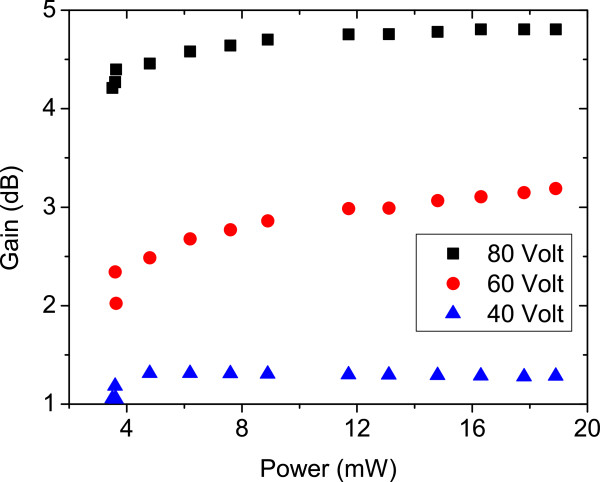
**Gain versus incident power using various applied voltages at *T*** **= 300 K.**

The spectra of EL, PL, and the combined EPL of bidirectional THH-VCSOA device at 1,280 nm are shown in Figure 6. The spectra have a broad bandwidth due to the fact that light was collected from the whole forward-biased area. The input signal of 488 nm is absorbed by the THH device, causing a modulation of the 1,280-nm light, thus acting as a wavelength converter. In EPL, the device is optically but also electrically pumped, with *V*_app_ = 80 V in amplitude. The EL spectrum alone was also measured with *V*_app_ = 80 V and the difference between EL + PL and EPL intensities is accountable for the gain from the device. Optical gains versus incident powers at various applied voltages are depicted in Figure 7. At *T* = 300 K, maximum gains of around 1.3, 3.3, and 5 dB at *V*_app_ = 40, 60, and 80 V, respectively, are observed.

## Conclusions

The operation of bidirectional THH-VCSOA-based Ga_0.35_In_0.65_ N_0.02_As_0.08_ at a wavelength of 1,280 nm has been demonstrated. Maximum optical gain of about 5 dB is observed at *V*_app_ = 80 V and at *T* = 300 K. Therefore, we conclude that the THH-VCSOA device is a bidirectional field-effect light-emitting and light-absorbing heterojunction and can work as an optical amplifier and wavelength converter in the 1.3-μm wavelength regime. The performance of the device can be improved by reducing the dimensions of the device, so that high electrical fields can be reached by the application of small voltages.

## Abbreviations

DBRs: distributed Bragg reflectors; EL: electroluminescence; EPL: electrophotoluminescence; HELLISH: hot electron light-emitting and lasing in semiconductor heterostructure; MBE: molecular beam epitaxy; PL: photoluminescence; THH: Top-Hat HELLISH; QWs: quantum wells; VCSOA: vertical-cavity semiconductor optical amplifier.

## Competing interests

The authors declare that they have no competing interests.

## Authors’ contributions

NB and FAIC designed the structure. FAIC fabricated the devices and carried out the experimental work and wrote the article. NB is the inventor of the original device and the overall supervisor of the project. Both authors read and approved the final manuscript.
